# ARBIN: Augmented Reality Based Indoor Navigation System

**DOI:** 10.3390/s20205890

**Published:** 2020-10-17

**Authors:** Bo-Chen Huang, Jiun Hsu, Edward T.-H. Chu, Hui-Mei Wu

**Affiliations:** 1 Department of Computer Science and Information Engineering, National Yunlin University of Science and Technology, Yunlin 64002, Taiwan; m10717004@yuntech.edu.tw; 2 National Taiwan University Hospital YunLin Branch, Yunlin 640203, Taiwan; Y00051@ms1.ylh.gov.tw (J.H.); Y01614@ms1.ylh.gov.tw (H.-M.W.)

**Keywords:** augmented reality, Bluetooth, indoor positioning system, indoor navigation system, smart hospital

## Abstract

Due to the popularity of indoor positioning technology, indoor navigation applications have been deployed in large buildings, such as hospitals, airports, and train stations, to guide visitors to their destinations. A commonly-used user interface, shown on smartphones, is a 2D floor map with a route to the destination. The navigation instructions, such as turn left, turn right, and go straight, pop up on the screen when users come to an intersection. However, owing to the restrictions of a 2D navigation map, users may face mental pressure and get confused while they are making a connection between the real environment and the 2D navigation map before moving forward. For this reason, we developed ARBIN, an augmented reality-based navigation system, which posts navigation instructions on the screen of real-world environments for ease of use. Thus, there is no need for users to make a connection between the navigation instructions and the real-world environment. In order to evaluate the applicability of ARBIN, a series of experiments were conducted in the outpatient area of the National Taiwan University Hospital YunLin Branch, which is nearly 1800 m^2^, with 35 destinations and points of interests, such as a cardiovascular clinic, x-ray examination room, pharmacy, and so on. Four different types of smartphone were adopted for evaluation. Our results show that ARBIN can achieve 3 to 5 m accuracy, and provide users with correct instructions on their way to the destinations. ARBIN proved to be a practical solution for indoor navigation, especially for large buildings.

## 1. Introduction

Due to the advance of the internet of things and business opportunities, indoor navigation systems have been deployed in many large buildings, such as big train stations, shopping malls, hospitals, and government buildings. After installing a navigation mobile app, users can select a point of interest on a menu list. Then, the app will determine a route to the destination, which is usually the shortest path. Nowadays, the most commonly used user interface (UI) of navigation applications is a 2D map with a route. Users are provided with navigation instructions, such as turn left, turn right, and go straight, when they are close to an intersection. However, due to the limitations of a 2D navigation map, it could add an additional cognitive load for users to construct the relationship between the 2D navigation map and the real environment. Extra mental pressure may also be induced and make users confused [1]. Therefore, eliminating possible user confusion is important for navigator UI design.

In order to create a good user experience, several research efforts have been devoted to developing an indoor navigation system by utilizing augmented reality (AR) technology. A. Mulloni et al. [2,3] and L. C. Huey et al. [4] deployed markers as location anchors in the environment. A user can know their location by matching the markers with the associated location information stored at a remote server or on a user’s phone. However, the angle of the camera must be in proper alignment with markers before the matching process can start. In addition, the markers could get dirty easily and become unrecognizable, therefore increasing maintenance costs. S. Kasprzak et al. [5], J. Kim et al. [6] first performed an image search for pre-tagged objects, such as billboards and trademarks, in the environment, and then determined the user’s location based on the obtained objects. However, the more complicated the environment is, the more difficult it will be to identify pre-tagged objects. The image matching processing becomes even challenging when the layout and decoration of different parts of the space are similar. Feature matching is another method to determine user’s location [1]. However, constructing point clouds of a real indoor environment is time consuming and costly, especially for a large building.

In this paper, we designed ARBIN, an augmented reality-based navigation system, by extending our previous work, WPIN [7]. WPIN utilized Bluetooth low energy (BLE) beacons, named Lbeacons version 1 (BiDaE Technology, Taipei, Taiwan), deployed at each intersection and point of interest (POIs), to get the coordinates of the current position. 2D images, such as turn left, turn right, and go straight, were provided to users as direction indicators along the route to the destination. Unlike WPIN, ARBIN uses AR technology that combines virtual objects and the real world. Navigation instructions, as well as AR 3D models, are posted on the screen on the surrounding environment through the smartphone camera. Therefore, there is no need for users to make a connection between the navigation instructions and the real-world environment. In our implementation, Google ARCore (Google Inc., Mountain View, California, United States) [8] is adopted to create AR 3D models, obtain gyroscope sensor readings, and determine where to put the models. Accuracy is the key factor for the success of an AR-based indoor navigator. The difficulties in achieving accuracy of indoor positioning, and that of AR 3D model placement are described as follows.

In WPIN [7], Lbeacons were adopted at waypoints to periodically broadcast their own coordinates to smartphones nearby. A waypoint can be an intersection, a point of interest (POI), or the middle of a corridor. After receiving a broadcast message sent from a Lbeacon, the positioning module, running on the user’s smartphone, starts to estimate the distance between itself and the Lbeacon according to a RSSI (received signal strength indicator) distance model. The stronger the received signal is, the closer the user is to the Lbeacon. When the user and the Lbeacon are close enough, for example less than 5 m, a new direction indicator will pop up to guide the user to the next waypoint. The above process continues until the user arrives the destination. However, because of the machine cutting error, the size of the antenna board of each Lbeacon may not be identical, which could affect its capability for transmitting and receiving signals. Furthermore, the characteristics of the RF (Radiofrequency) circuit of each Lbeacon may also be different due to the nature of an analogy circuit. Therefore, the RSSI distance model of each Lbeacon is not exactly identical according to our experience. In our previous work, to achieve the required positioning accuracy, we constructed a RSSI model for each Lbeacon, which was time consuming and unscalable. To overcome this unavoidable and challenging hardware problem, a novel RSSI modeling method was developed to overcome the problem of the heterogeneous issues of Lbeacons, which is given in Section 3.2.

The AR 3D models, such as a left arrow or a right arrow, should be placed properly in a real-world environment to avoid possible user confusion. An inaccurate placement of the 3D model may make users confused, and avoid using it. For example, displaying a 3D model in the wrong orientation, an incorrect elevation angle, or an incorrect depression angle. Many parameters should be carefully considered before having the correct placement of a 3D model, such as the face orientation of a user, the location, and orientation of the smartphone. Constructing a relationship between these parameters and the coordinates of a 3D model is challenging. The detailed method is presented in Section 3.4 to Section 3.5.

In order to investigate the applicability of ARBIN, we first evaluated the responsiveness of the positioning module of ARBIN. We then set up a field trial in a hospital. For the former, we conducted a series of experiments in the engineering building No. 5 of the National Yunlin University of Science and Technology, crossing three floors with a total area of around 250 m^2^. The experiment results showed that the adopted RSSI (received signal strength indicator) model could accurately determine the distance between a Lbeacon and a smartphone. Thus, the AR models could be displayed correctly on the smartphone screen. Furthermore, a field trial was conducted at the outpatient area of the National Taiwan University Hospital YunLin Branch, which is nearly 1800 m^2^, with 35 destinations and point of interests, such as a cardiovascular clinic, x-ray examination room, pharmacy, and so on. Four different types of smartphone were adopted for evaluation. Our results show that ARBIN can achieve 3 to 5 m accuracy and give users correct instructions on their ways to the destinations. ARBIN proved to be a practical solution for indoor navigation, especially for large buildings.

## 2. Related Work

Due to the promise of providing a user-friendly interface to users, several researchers have utilized AR technologies to develop indoor navigation applications. Based on the positioning technologies they used, the existing AR-based navigations can be classified into three types: marker-based method, 2D image recognition-based method, and 3D space recognition-based method. Each of them is described as follows.

### 2.1. Marker-Based Methods

For marker-based methods, markers are first deployed in an indoor environment, and function as location anchors. A marker can be a QR-Code or a specially-designed pattern. The universally unique identifier (UUID) and coordinate of each marker are pre-stored in either the local storage of a smartphone or a remote database for future queries. When finding a marker, the user points the smartphone camera at the marker and scans it. The scanned image is then used for determining the user’s location and the place to put a 3D AR model. A. Mulloni et al. [2,3], L. C. Huey et al. [4], G. Reitmayr et al. [9], F. Sato [10], and C. Feng et al. [11] used highly recognizable pictures as markers. Each marker is regarded as a node of the navigation route. When a user comes to a marker and aims the camera at it, a 3D arrow model will be shown on the screen of smartphone to guide the user to the next node. Although markers are easy to deploy, extra user training may be needed to make a marker-based navigation system successful. For example, users should be able to identify markers in the surrounding environment before getting location information and moving forward. It is particularly difficult for a user who has no idea of what a marker looks like. In addition, the camera should be aligned with the marker to ensure the correctness of marker recognition, which makes it user-unfriendly. Maintenance could also be a critical issue when markers get dirty and become unrecognizable. Owing to the above limitations, marker-based navigation is not considered in our work.

### 2.2. 2D Image Recognition-Based Methods

Unlike marker-based methods, 2D image recognition-based methods search for pre-annotated objects, such as billboards, trademarks, and signs in the environment, and then determine the user’s location based on the obtained objects [5,6,12,13,14,15]. With the development of image recognition technology, there is a trend for replacing marker-based navigation with image recognition-based navigation. Although the image recognition-based methods may not have the problem of maintenance, they could fail when two objects are similar and undifferentiable. For example, in a large shopping mall, chairs, signboards, and decorations are usually designed in a similar way, which makes it difficult for image recognition. Many other factors can also affect the accuracy of image recognition, such as the view angle of the camera, the distance between the objects and the user, the number of moving objects in the surrounding environment, and so on. Since all these issues should be well addressed before an indoor navigation system can well function in a large and crowded building, we decided not to use an image recognition-based method. Both G. H. Nam et al. [16] and J. Wu et al. [17] adopted image recognition technology for indoor navigation. However, many factors can also affect the accuracy of image recognition, such as the view angle of camera, the distance between the objects and the user, the number of moving objects in the surrounding environment, and so on.

### 2.3. 3D Space Recognition-Based Methods

Compared to 2D image recognition-based methods, 3D space recognition-based methods collect features of the entire space rather than the features of 2D objects. During runtime, a feature matching mechanism is first used to determine a user’s location, and then an AR engine is adopted to draw a 3D model for direction indication. For the implementation, G. Gerstweiler et al. [18], U. Rehman et al. [1] used Metaio SDK (Metaio, Munich, Germany) [19] to create a 3D point cloud for indoor positioning. T. Rustagi et al. [20] used the MapBox API (Mapbox, San Francisco, California, United States) [21] to collect vector data of the indoor environment, and create a corresponding 3D model. The Unity (Unity Technologies, San Francisco, California, United States) [22] game engine was adopted in their system to show AR models. Although 3D could deliver better indoor positioning accuracy, construing 3D models is costly and time consuming, especially for large buildings. In addition, re-modeling is required when indoor layout is changed, which usually happens to shopping malls and exhibition halls. Furthermore, moving objects inside the space could significantly reduce positioning accuracy. The more crowded the environment is, the lower positional accuracy will be. A. Koc et al. [23] adopted ARKit (Apple Inc., Cupertino, California, United States) [24] to build an AR-based indoor navigation. Similar to ARCore, ARKit constructs an AR world with associated 3D information. However, the proposed method requires the user to create coordinate information of all corners in the indoor environment, which makes it time consuming and error prone. In addition, their experimental results showed that the accumulated error becomes significant when the place is large. H. Choi et al. [25] proposed a virtual anchor (VA) point selection method for AR-assisted sensor positioning. According to their definition, a VA is a positioning reference point used by a UWB (Ultra-Wideband) positioning mechanism. The more VAs are selected, the more time is required to determine the user’s location. Since UWB devices are required, the issues of hardware cost and energy consumption should also be addressed before a deployment.

Unlike existing feature-matching methods, ARBIN utilized Lbeacons deployed at each intersection and POIs to get the coordinates of the current position. ARBIN performs well in crowded spaces due to the advantage of direction antenna built into Lbeacons, which can adjust transmission power and beam width to properly cover navigation areas. Finally, ARBIN is easy to configure and maintain.

## 3. Methodology

### 3.1. System Overview

**Figure 1:**
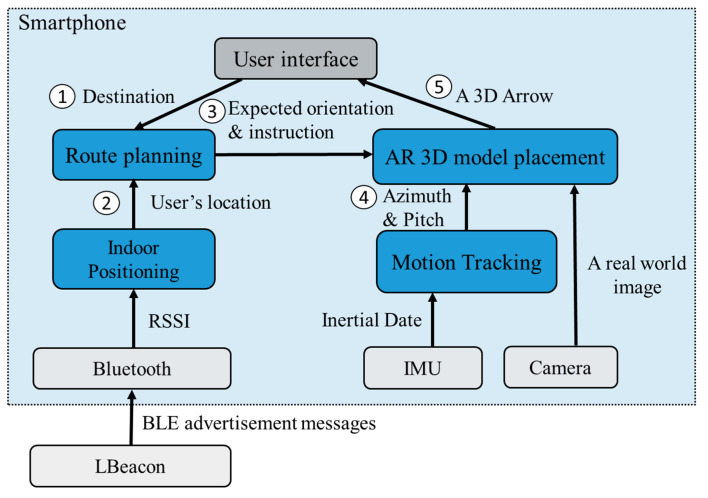
System architecture of ARBIN.

As Figure 1 shows, ARBIN consists of four modules: indoor positioning, route planning, motion tracking, and AR 3D model placement. At the beginning, the destination selected by the user is sent to the route planning module for determining of a route to the destination (Step 1). The underneath indoor positioning module continuously updates the user’s location based on the received BLE advertisement messages and the associated RSSI (Step 2). When the user comes to a waypoint, the route planning module sends a message including the expected face orientation and directional indicator to the AR placement module (Step 3). The AR placement relies on the motion tracking module to obtain the direction (azimuth) and the pitch of the smartphone from the IMU (Inertial Measurement Unit) (Step 4). Based on the collected information, the placement module overlays a 3D arrow model, such as turn left or turn right, on the real-world image (Step 5).

**Figure 2:**
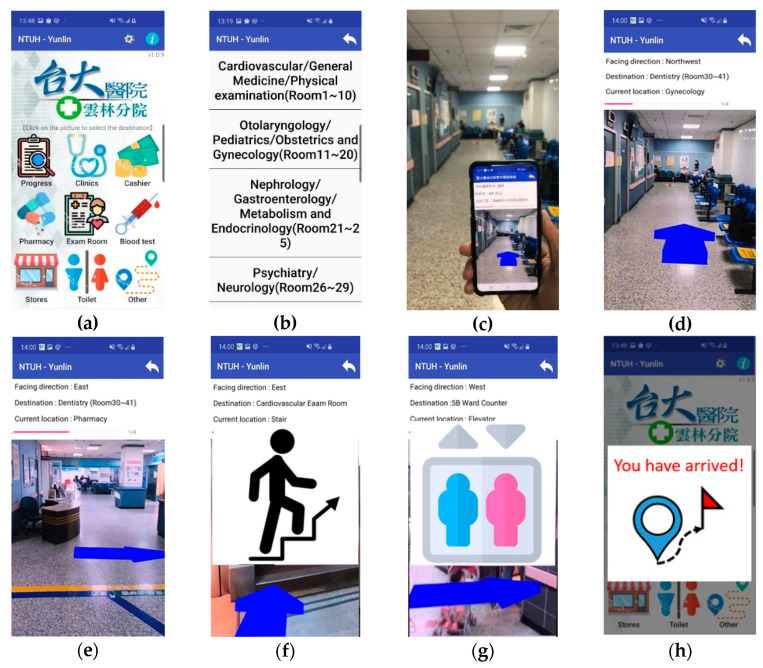
The user interface of ARBIN. (**a**) Main page of ARBIN; (**b**) Destination list; (**c**) Start navigation service; (**d**–**g**) 3D indicator of navigation instruction; (**h**) Arrival message.

Figure 2 lists the user interface of the ARBIN App. Frequently asked destinations are shown on the main page (Figure 2a). After a user selects a destination on the list (Figure 2b), ARBIN determines the user’s current location and a route with the shortest distance to the destination. At the beginning, the user is asked to face a specific direction before the navigation service starts (Figure 2c). In other words, the navigation service will not start until the user faces the expected orientation. On the way to the destination, a 3D indicator will be placed in the real-world environment when the use approaches an intersection or a point of interest, such as stairs or elevators (Figure 2d–g). The navigation service stops when the user arrives at the destination. Finally, a message pops up to reminder the user that the navigation service is finished (Figure 2h).

### 3.2. Indoor Positioning Module

**Figure 3:**
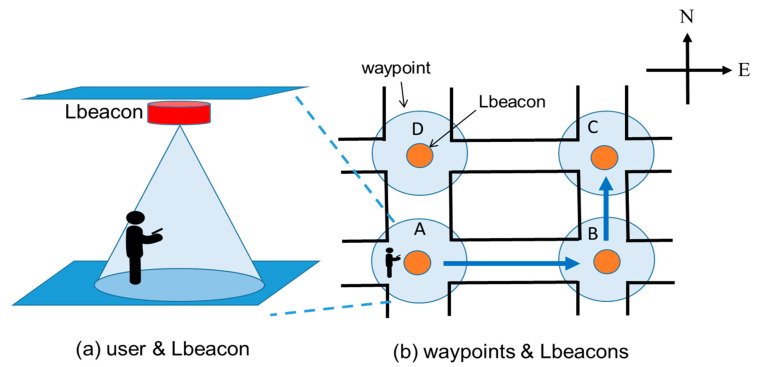
The positioning method of waypoint-based navigation [7]. (**a**) User and Lbeacon; (**b**) waypoint and Lbeacons.

The purpose of the positioning module is to determine the user’s location. As Figure 3a shows, Lbeacons are deployed at waypoints. In this work, a waypoint is defined as an intersection, a point of interest, or the middle of a corridor. Each Lbeacon periodically broadcasts its coordinate information to smartphones nearby. From the view point of the user, his or her smartphone continuously receives the coordinate information sent by Lbeacons nearby, while determining how far the smartphone is from the closest Lbeacon. If the distance between the smartphone and a Lbeacon is close enough, for example 3 m, the navigation app provides the user with a directional indicator to guide him or her to the next waypoint. An illustrated example is shown in Figure 3b, the route starts from waypoint A and ends at waypoint C. The user first receives a “go straight” command when entering the area of waypoint A, and then a “turn left” command at waypoint B. The coverage size of a waypoint depends on the size of the intersection or the point of interest. The larger the coverage area is, the larger the range of a waypoint is. In our implementation, the coverage size of a waypoint is a 3-m, 5-m, or 7-m radius circle. The key factor for waypoint-based navigation success is accurately determining the distance between the user and the Lbeacons. For this, in our previous work [7], RSSI distance models stored on the smartphone were adopted to estimate the distance. However, because of the machine cutting error and the characteristics of the RF circuit, the RSSI distance model of each Lbeacon is not identical. To achieve the required positioning accuracy, we constructed a RSSI model for each Lbeacon, but it was time consuming and unscalable.

**Figure 4:**
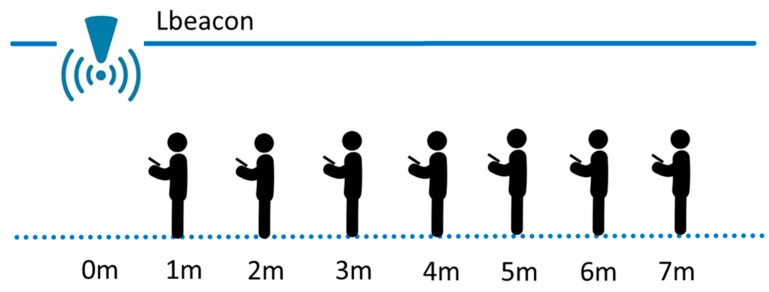
Collecting received signal strength indicator (RSSI) samples at different distances.

To overcome this problem, in this work we first analyzed the characteristics of RSSI models of about 24 randomly selected Lbeacons, from 70 Lbeacons. We then classified them into four types. For each type of Lbeacon, only one RSSI model was used. Because the navigator must give a user a directional indicator when he/she enters the coverage of a waypoint, we mainly focused on the behavior of the RSSI curve in the range of 0 to 3 m, 3 to 5 m, and 5 to 7 m. As Figure 4 shows, we measured the RSSI values at the locations 1 m to 7 m away from a Lbeacon. For each location, we collected one-minute of RSSI samples (i.e., 240 samples) and took the average as the result. The measurement stops when the seven locations have been measured.

**Figure 5:**
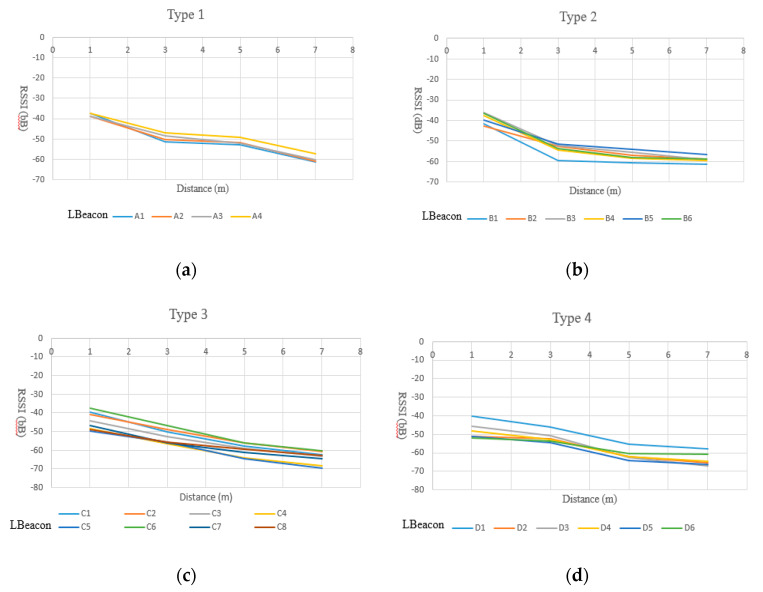
Four types of RSSI distance models. (**a**) Type 1; (**b**) Type 2; (**c**) Type 3; (**d**) Type 4.

**Figure 6:**
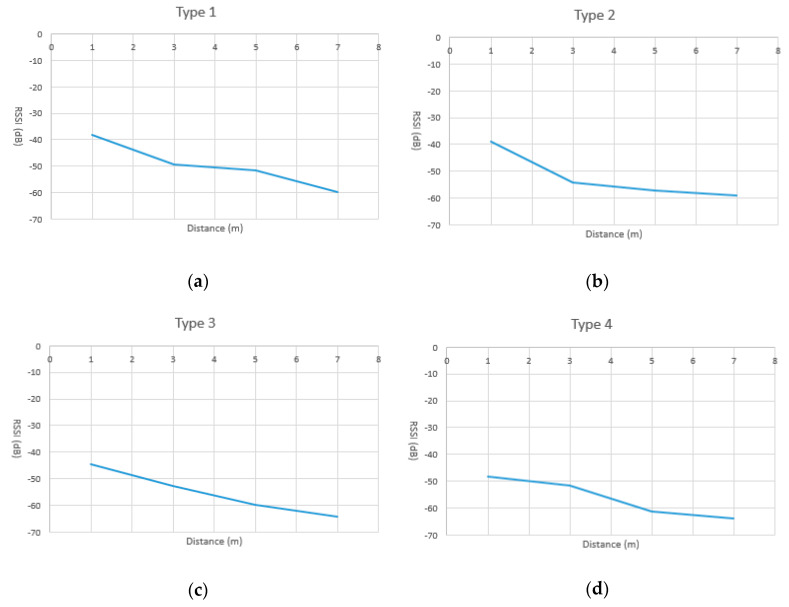
The regression model of the distance and the RSSI values. (**a**) The regression model of type 1 Lbeacon; (**b**) The regression model of type 2 Lbeacon; (**c**) The regression model of type 3 Lbeacon; (**d**) The regression model of type 4 Lbeacon;.

As shown in Figure 5a, four Lbeacons, numbered A1, A2, A3, and A4, were classified as type 1, in which the RSSI values drops inversely to the distance, in the range of 0–3 m and 5–7 m. Therefore, Type 1 Lbeacons are suitable to cover a waypoint with a radius of 3 m and 7 m. Similarly, Type 2 Lbeacon are only suitable to cover a waypoint with a radius of 3 m. Meanwhile, Type 3 Lbeacons are suitable for a waypoint with radius of 3, 5, or 7 m since the RSSI values drops inversely to all the distances we measured. Type 4 Lbeacons are suitable for a waypoint with a radius of 5 m. Based on the measurement, for each type of Lbeacon, we adopted a polynomial function as a regression model to represent the relationship between the distance and the RSSI values. Results are shown in Figure 6. Given a new and unknown type of Lbeacon, we first classified it into one of the four types based on the characteristic of its RSSI curve. A RSSI model was then picked from the RSSI models shown in Figure 6. In Section 5, the correctness of the proposed models shown in Figure 6 is evaluated.

To tolerate the variability of RSSI values, we considered the RSSI values of Lbeacons nearby. Let $S_{i}$ and $S_{j}$ represent the highest and the second-highest RSSI detected by the smartphone. The $S_{i}$ is the RSSI of waypoint i and the $S_{j}$ is that of waypoint, j. Since $S_{j}$ is the highest, the user is regarded as being at waypoint i. Based on the RSSI models, we can obtain the theoretical value of $S_{i}$ and $S_{j}$ at waypoint i; that is, ${\overset{´}{S}}_{i}$ and ${\overset{´}{S}}_{j}$. If $S_{i}$ – $S_{j}$ >= ${\overset{´}{S}}_{i}$ – ${\overset{´}{S}}_{j}$, the user’s location is updated to waypoint *i*. On the other hand, if $S_{i}$ – $S_{j}$ < ${\overset{´}{S}}_{i}$ – ${\overset{´}{S}}_{j}$, the *S_i_* is considered as a signal surge and will be filtered out.

All Lbeacons were classified into four types. As Figure 6a,d show, each type has its own RSSI model. The RSSI model used to determine the distance between a user’s smartphone and a Lbeacon depended on the type of the Lbeacon. When getting close to a Lbeacon, the smartphone uses received UUID to look up the type of Lbeacon and its associated RSSI model, pre-stored in the smartphone.

### 3.3. Route Planning Module

After receiving the information of user’s location and destination, shown in Figure 1, the route planning (RP) module determines a route to the destination by the well-known Dijkstra’s shortest path algorithm. Based on the route, the RP module updates the AR model placement module with a direction indicator and an expected face orientation when the user comes to a waypoint. The two pieces of information are then used for placing a 3D model on the real-world environment. For example, as Figure 3b shows, the user starts at waypoint A and moves to waypoint B. When the user enters the coverage of waypoint B, the expected face orientation is east. After the user turns left and moves forward, his/her expected face orientation at waypoint C is north. For ARBIN, at each waypoint, if the user’s orientation is not the same as the expected face orientation, the associated directional indicator will not show in the real-world environment. A warning message will pop up to remind the user, when needed. If this happens, possible reasons are that the user is going the wrong way, or that the user does not face to the expected orientation. The route will be recalculated if the user is found at an unexpected waypoint. In our implementation, the orientation is obtained by IMU (inertial measurement unit) sensors of the smartphone. ARBIN uses the getOrientation() of Android Sensor Manager [26] to obtain the orientation. In the above-mentioned example, if B and C are not detected when the user arrives at D, ARBIN will recalculate the route. Then D will be a new starting point.

**Figure 7:**
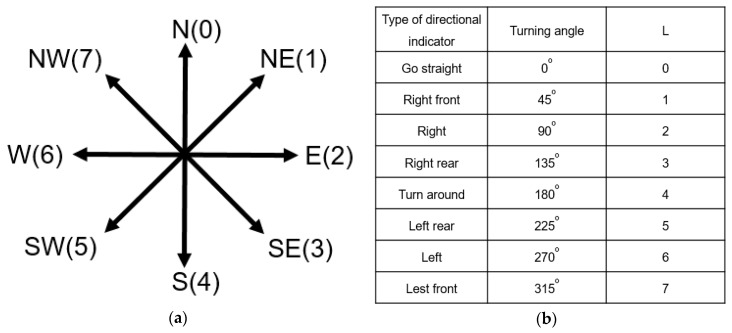
The definition of orientation and directional indicators. (**a**) R values; (**b**) L values.

Let *R* denote the expected orientation at a waypoint. The *R* is an integer between 0 to 7, each of which represents a type of orientation, shown in Figure 7a. For example, *R* = 1 is northeast while *R* = 2 is east. After the user passes through a waypoint, the *R* is updated by how many degrees the user turns to the new orientation. For example, for a turn right instruction, the *R* is updated by adding 90°. Additionally, for a turn left instruction, the *R* is updated by adding 270°. Since there are only 8 types of directional indicator in our implementation, we use *L*, an integer between 0 to 7, to represent the turning angle of a directional indicator. The definition of each value of *L* is given in Figure 7b. When the user enters a waypoint, *R* is updated by (*R* + *L*) mod 8. For example, in Figure 3b, at the beginning, the user faces to the east and *R* is 2. When the user comes to waypoint B, the expected face orientation is east. After the user turns left and moves forward, the expected face orientation *R* at the waypoint C is updated to 0 (= (2 + 6) mod 8), which is north.

### 3.4. Motion Tracking Module

**Figure 8:**
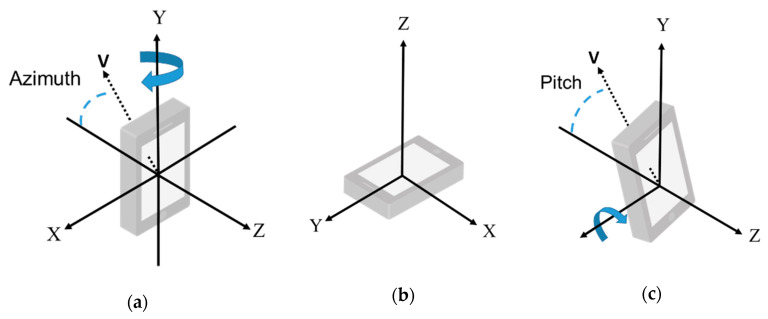
The orientation of a smartphone. (**a**) Phone is keeping upright; (**b**) Phone is lying flat; (**c**) The definition of pitch.

The motion tracking module aims to determine the direction (azimuth) and the pitch of the smartphone based on the magnetic sensor and the acceleration sensor of a smartphone. Since the coordinate system of the smartphone and earth are different, transformation is needed before the sensor readings can be used. As shown in Figure 8a, in our usage scenario, the smartphone should be kept upright so that a 3D model can be properly put onto a real environment. If the smartphone is laid flat, shown in Figure 8b, a warning message will be provided to remind the user. Let vector *V* be the heading direction of the smartphone. As Figure 8a shows, *V* is a vector on the *X-Z* plane. ARBIN uses the orientation of *V* as the expected face orientation. Moreover, the pitch of the smartphone should be greater than 80° before a 3D model can be displayed. The definition of pitch is shown in Figure 8c.

Let X, Y, and Z represent the three axes of the smartphone coordinate system S. In addition, $\overset{´}{X}$, $\overset{´}{Y},$ and $\overset{´}{Z}$ represent those of the earth coordinate system *G*. The $\theta_{ij}$ is the angle between the i axis of *S* and the j axis of *G*, in which i = X, Y, or Z, and j = $\overset{´}{X}$, $\overset{´}{Y}$, or $\overset{´}{Z}$. The angles can be obtained by the IMU sensor built into a smartphone. Let (x, y, z) be a point on S and its associated coordinate in *G* be $\left(\overset{´}{x},\;\overset{´}{y},\;\overset{´}{z}\right)$. Therefore, we have

$$\overset{´}{x}=\;x\cos\theta_{x\overset{´}{x}}+\;y\cos\theta_{x\overset{´}{y}}+z\cos\theta_{x\overset{´}{z}},$$

$$\overset{´}{y}=\;x\cos\theta_{y\overset{´}{x}}+\;y\cos\theta_{y\overset{´}{y}}+z\cos\theta_{y\overset{´}{z}},$$

$$\overset{´}{z}=\;x\cos\theta_{z\overset{´}{x}}+\;y\cos\theta_{z\overset{´}{y}}+z\cos\theta_{z\overset{´}{z}}.$$

The $\left(\overset{´}{x},\;\overset{´}{y},\;\overset{´}{z}\right)$ can be represented by ${\left(\overset{´}{x},\;\overset{´}{y},\;\overset{´}{z}\right)}^{T}=R\left(x,y,z\right)$. The R the rotation matrix which is:

$$R=\left(\begin{array}{ccc} \cos\theta_{x\overset{´}{x}} & \cos\theta_{x\overset{´}{y}} & \cos\theta_{x\overset{´}{z}}\\ \cos\theta_{y\overset{´}{x}} & \cos\theta_{y\overset{´}{y}} & \cos\theta_{y\overset{´}{z}}\\ \cos\theta_{z\overset{´}{x}} & \cos\theta_{z\overset{´}{y}} & \cos\theta_{z\overset{´}{z}} \end{array}\right).$$

Therefore, when the smartphone has a rotation around different axis, we can have a different rotation matrix [27]. They are:

$$R_{P}=\left(\begin{array}{ccc} 1 & 0 & 0\\ 0 & \cos\theta_{y\overset{´}{y}} & \cos\theta_{y\overset{´}{z}}\\ 0 & \cos\theta_{z\overset{´}{y}} & \cos\theta_{z\overset{´}{z}} \end{array}\right)=\left(\begin{array}{ccc} 1 & 0 & 0\\ 0 & \cos P & \sin P\\ 0 & -\sin P & \cos P \end{array}\right),$$

$$R_{A}=\left(\begin{array}{ccc} \cos\theta_{x\overset{´}{x}} & 0 & \cos\theta_{x\overset{´}{z}}\\ 0 & 1 & 0\\ \cos\theta_{z\overset{´}{x}} & 0 & \cos\theta_{z\overset{´}{z}} \end{array}\right)=\left(\begin{array}{ccc} \cos A & 0 & -\sin A\\ 0 & 1 & 0\\ \sin A & 0 & \cos A \end{array}\right),$$

$$R_{O}=\left(\begin{array}{ccc} \cos\theta_{x\overset{´}{x}} & \cos\theta_{x\overset{´}{y}} & 0\\ \cos\theta_{y\overset{´}{x}} & \cos\theta_{y\overset{´}{y}} & 0\\ 0 & 0 & 1 \end{array}\right)=\left(\begin{array}{ccc} \cos0 & \sin0 & 0\\ -\sin0 & \cos0 & 0\\ 0 & 0 & 1 \end{array}\right),$$

in which $R_{P}$ is the rotation matrix when the smartphone has a rotation around X axis, and P is the pitch angular. In addition, $R_{A}$ is the rotation matrix when the smartphone has a rotation around Y xis, and A is the azimuth angular. Furthermore, $R_{O}$ is the rotation matrix when the smartphone has a rotation around the *Z* axis and O is the roll angular. By using the rotation matrix and rotation angles, we can transform a coordinate between S and G.

In our implementation, the Android sensor manager (Google Inc., Mountain View, California, United States) [26] is adopted to transfer the V vector from the smartphone coordinate system, S, to the earth coordinate system, G, and obtain the pitch of the smartphone. ARBIN invokes the getRotationMatrix() function to get a rotation matrix, by feeding the sensor readings of the magnetic sensor and the acceleration sensor. The rotation matrix transformation is used to transform the vectors and coordinates from the smartphone coordinate system to the earth coordinate system. Based on the rotation matrix, ARBIN then uses getOrientation() to obtain the orientation, azimuth, and pitch of the smartphone. Environment noises could affect the correctness of the IMU of the smartphones. For this reason, ARBIN can be integrated with advanced noise filters or probability models to reduce the interference. Since sensor calibration and compensation is not the major focus of this work, for the readers who are interested in this topic, please refer to [28,29,30].

### 3.5. AR 3D Model Placement Module

The purpose of the AR 3D model placement module (APM) is to overlay a 3D model on a real-world image. The process includes three steps: pitch check, face orientation check, and placement. Each of the steps is descried as follows. First, APM checks if the smartphone is kept upright. The larger the pitch angle is, the better the camera view is. In our implementation, the pitch angle is set in the range of 80 to 90°. If the pitch angle does not meet the requirement, a warning message is displayed to remind the user to adjust the pitch angle of the smartphone. Second, APM examines whether the orientation of the smartphone is the same as the expected face orientation. If both the pitch angle and the orientation of the smartphone meet the required conditions, a 3D model is placed onto a real environment.

**Figure 9:**
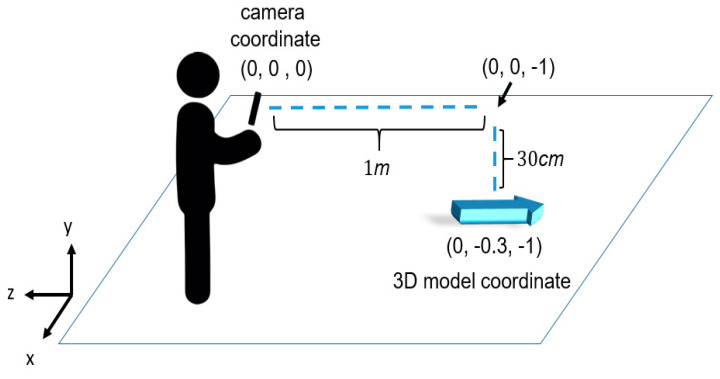
The coordinates of a 3D model.

The 3D model placement relies on visual-inertial odometry (VIO), which first uses a camera to extract special feature points of the surrounding environment, such as the corners, boundaries, and blocks. It then continuously matches the features in the contiguous frames to estimate the movement of the camera. Based on the movement of the camera, the 3D model can then be kept at the place we expected until the 3D model is not in the field of view of a camera. In our implementation, we used ViroCore SDK (Viro Media, Inc., Seattle, Washington, United States) [31] to implement the model placement module. ViroCore is a tool package built on top of AndroidARCore (Google Inc., Mountain View, California, United States) [8]. We used getLastCameraPositionRealtime () to get the camera coordinates, and getLastCameraForwardRealtime () to get the camera shooting direction. The calibration of camera depth is done by the smartphone itself. In our configuration, the 3D model is placed at 1 m away from the camera along the camera shooting direction. To have a better view, the 3D model is further put 30 cm below the camera shooting direction. For example, as Figure 9 shows, the camera coordinate is (0, 0, 0) and the camera shooting direction vector is (0, 0, –1). Taking the above-mentioned coordinates, ARBIN determines the coordinated 3D model by (0, 0, 0) + (0, 0, –1) + (0, –0.3, 0) = (0, –0.3, –1), in which the unit is meter. The ARCore then takes (0, –0.3, –1) as input and adopts VIO technology to places the 3D model in the place we expect.

## 4. Experiment

Our experiment included two parts: in-house experiments and a field trial. The in-house experiments were undertaken in the Engineering Building (EB) No. 5 of Yunlin University of Science and Technology (Yuntech). The purpose was to evaluate the orientation determination of a smartphone, and the correctness of the RSSI model proposed in Section 4.2. After the in-house experiments were completed, we then conducted a field trial at the National Taiwan University Hospital YunLin Branch (NTUH-Yunlin) where 35 Lbeacons were deployed in the outpatient area, over 1800 m^2^, covering two floors. Volunteers were invited to evaluate the responsiveness of ARBIN.

### 4.1. In-House Experiments

#### 4.1.1. Evaluation of Azimuth of Smartphones

ARBIN relies on information of face orientation to correctly place a 3D model in the real-world environment. Therefore, it is important to ensure that the azimuth value provided by a smartphone is correct. According to the Android sensor manager [26], azimuth is the angle between the smartphone’s current compass direction and magnetic north. If the smartphone faces magnetic north, the azimuth is 0°; if it faces south, the azimuth is 180°. additionally, if it faces west, the azimuth is 270°, and if it faces east, the azimuth is 90°.

In our experiment, we investigated two selected smartphones, shown in Table 1, and checked if they could determine the azimuth correctly. We tested all possible orientations used by ARBIN. They are north, northeast, east, southeast, south, southwest, west, and northwest. For each direction, we kept the smartphones upright and recorded the readings of azimuth provided by the smartphone for 10 s. The average value was taken for evaluation. As Table 2 shows, both the Samsung S10e (Samsung, Seoul, South Korea) and SONY Xperia XZ premium (Sony, Tokyo, Japan) could achieve a percentage error less than 5° in determining the azimuth. The ground truth of each measurement was obtained by a real compass. For example, the azimuth error of Samsung S10e when it faces north ranged from +2.66 to –3.32°. The maximum error was 4.67° when it faced Northeast. The larger the azimuth error is, the higher is possibility it will make the user confused. According to our experience, a user’s maximum tolerance level is 20°. The azimuth errors of the two smartphones were small enough to be tolerated, and will not affect the placement of a 3D model in a real-world environment. No further calibration on the azimuth angle was required.

#### 4.1.2. Responsiveness of ARBIN

This experiment investigated the ability of ARBIN to have a proper reaction when a user comes close to a Lbeacon. In order to provide a good user experience, we defined 3 m as the responsiveness distance. The ARBIN needs to provide the user with a directional indicator when he or she enters the area of a circle with radius of 3 m, where the Lbeacon is at the center of circle. In other words, it is meaningless to notify the user when he or she is not in the area, because the distance between the user and the Lbeacon is too far away. Similarly, notifying the user after he or she has already passed through the Lbeacon is also useless. The better the responsiveness of ARBIN, the better the user experience we create. The results of responsiveness can also represent the correctness of the four RSSI models presented in Section 4.2, because ARBIN relies on the four models to estimate the distance between the user and a Lbeacon.

**Figure 10:**
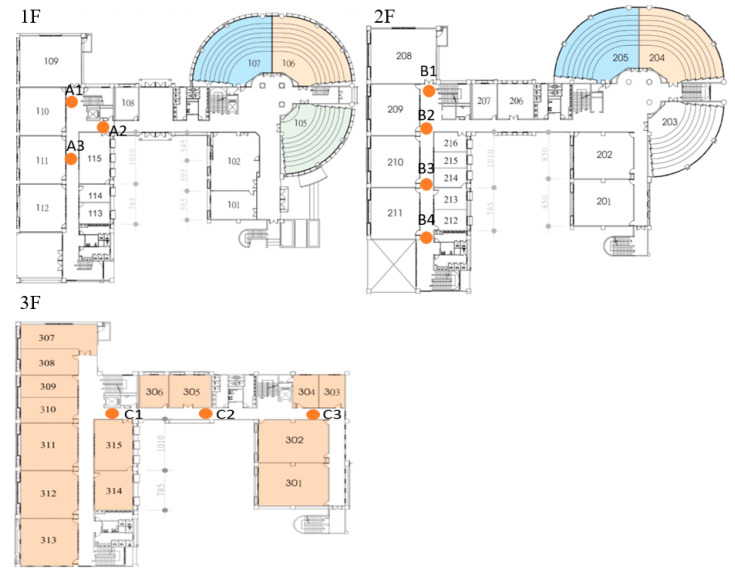
The deployment maps of Lbeacons in EB-No. 5 at Yuntech.

The in-house experiment was conducted in EB-No.5 of Yuntech, where 10 Lbeacons were deployed in three floors, with around 250 m^2^. Figure 10 shows the deployment maps. The height of the ceiling was about 3 m. When a user holds a smartphone for indoor navigation, the distance between the smartphone and the ground (i.e., 1.5 m) is almost the same as the distance between the smartphone and the ceiling. Hence, there is no significant difference in RSSI by putting Lbeacons on the ground or mounting them on the ceiling according to our experiment. To have a quick deployment without affecting the interior decoration, Lbeacons were put on the ground for the in-house experiment.

**Figure 11:**
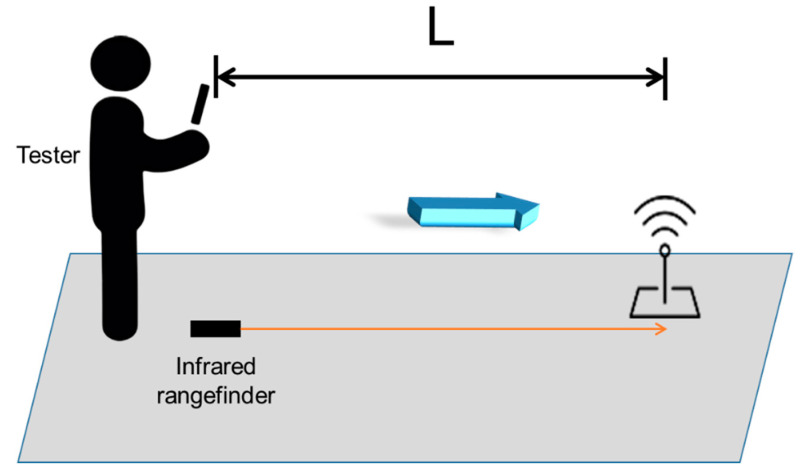
The experimental setup for measuring responsiveness.

**Figure 12:**
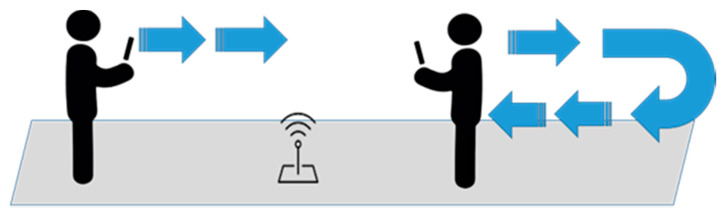
The process of measuring the responsiveness.

Figure 11 shows the experiment setup for measuring responsiveness. The tester walked around in the building with a normal speed of about one meter per second. As Figure 12 shows, for each Lbeacon, the tester walked back and forth five times. When receiving the directional indicator indicated by ARBIN, he stopped and measured the responsiveness distance, L, between the smartphone and the Lbeacon. As Figure 11 shows, the L is measured by an infrared rangefinder. In other words, for each Lbeacon, the tester first walked forward to the Lbeacon, then recorded the L after he was notified by ARBIN’s directional instruction. Then, the tester kept walking. After leaving the range of the Lbeacon (i.e., a circle with radius of 3 m), he turned around and walked forward to the Lbeacon again from the opposite direction. The same measurement was conducted again when the tester was close to the Lbeacon. For each Lbeacon, the tester repeated the above-mentioned experiment for five rounds. The average values of L in both the forward direction and backward direction are shown in Table 3. For the Lbeacons placed on a north-south corridor, the forward direction pointed to north, and the backward direction pointed to south. In addition, for the Lbeacons deployed on an east-west corridor, the forward direction pointed to east, and the backward direction pointed to west.

In our in-house experiment, the responsiveness distance should have been less than 3 m in order to create a good user experience. As Table 3 shows, in 92.5% (=37/40) of the test cases, ARBIN could properly notify the tester when the tester was close to a Lbeacon. However, there was an exception at Lbeacon A3. When the tester held a Samsung Galaxy smartphone and moved forward to Lbeacon A3, the smartphone notified the tester earlier than was expected. In other words, the smartphone received a relatively strong signal when it was 5 m away from Lbeacon A3. A possible solution was to slightly raise the software threshold of RSSI for Lbeacon A3 to defer the notification. We also found that the responsiveness distance, L, of the same Lbeacon differed the in forward and backward directions. The possible reason is that the directional antennas of the Lbeacons may have different abilities to send out signals in different directions. Although the responsiveness distance may depend on the user’s arrival direction, it does not affect the ability of ARBIN, in providing users a proper directional indicator. Furthermore, the results showed that there were no cases of notifying the tester after he had already passed the Lbeacon, which met our requirements.

The Lbeacon is equipped with a directional antenna with conical beams [32]. It can generate a 3 m range and 60° radiation pattern to provide a 3 m horizontal accuracy. However, according to our experience, the shape of the conical beams is not as perfect as is claimed. Therefore, the responsiveness distance, L, of the same Lbeacon may differ in forward and backward directions.

### 4.2. Field Trial

**Figure 13:**
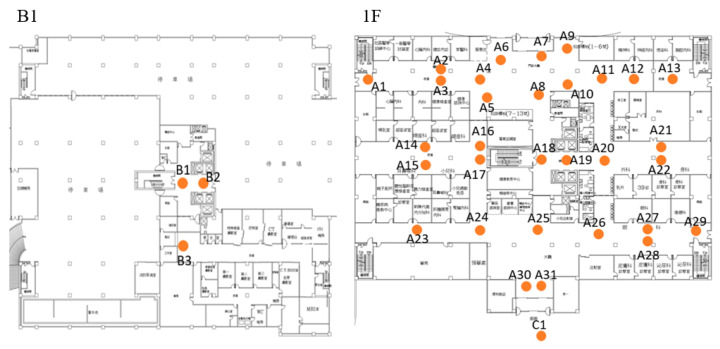
The deployment maps of Lbeacons in NTUH-Yunlin.

**Figure 14:**
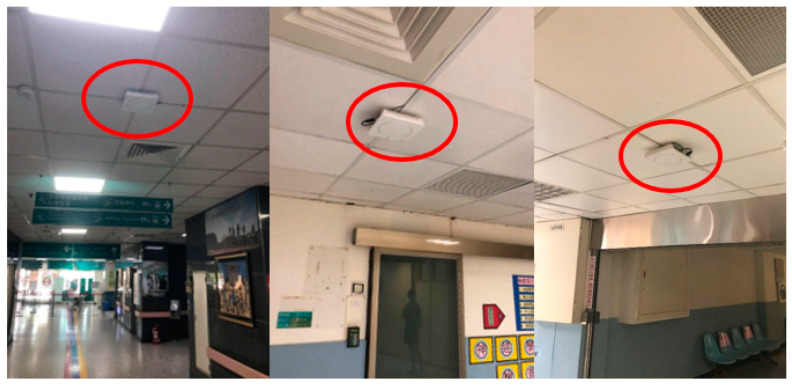
The installation of Lbeacons in the outpatient area of NTUH-Yunlin.

The purpose of the field trial was to evaluate the responsiveness of ARBIN in NTUH-Yunlin by collecting user’s feedback. The deployment maps are shown in Figure 13, in which 35 Lbeacons were deployed in the outpatient area (i.e., B1 and 1F) of the new medical building. Figure 14 shows the installation of Lbeacons mounted on the ceiling. Each Lbeacon periodically broadcasted its UUID at 4 Hz to the smartphones nearby. The starting point was unknow to ARBIN. Based on the UUID the smartphone received, ARBIN automatically determined the starting point. We invited four volunteers, with an average age of 25, who had never either used ARBIN or been to the hospital. They were asked to judge the responsiveness of ARBIN whenever they arrived at a waypoint. The acceptable responsiveness distance was set at 5 m because the coverage area of a Lbeacon deployed in the hospital was a circle with radius of around 5 m. If the ARBIN notified a volunteer after he or she entered the coverage range of a Lbeacon, the responsiveness was moderate. Other hand, if the ARBIN notified a user before he or she entered the coverage range (i.e., the responsiveness distance was larger than 5 m), the responsiveness was fast. In addition, the responsiveness was slow when the responsiveness distance approached zero. The above standards were told to the volunteers before they started the testing. A calibration was required for a new smartphone. Users were required to stand at a specific location, for example the entrance of the building, for 5 to 10 sec. ARBIN will automatically adjust thresholds based on the received RSSI values. Since the purpose of field trial was to evaluate the user experience, volunteers judged the responsiveness visually rather than using an infrared range finder. To simulate an outpatient flow, the volunteers were asked to go to the following destinations accordingly: registration counter (A11), X-ray examination room (B3), pharmacy (A25), and exit (C1). Detailed information of each route and the Lbeacons on that route are listed in Table 4.

As Table 4 shows, each volunteer passed through nine Lbeacons on their way to the destinations. They judge the responsiveness whenever they reach a waypoint. The results show that 97% (35/36) of the user feedbacks were “moderate”, which indicates that ARBIN can properly notify users when they approach a waypoint. Volunteer C marked A18 as slow because he experienced a deferred notification when he approached A18. A possible solution could be slightly decreasing the threshold of A18 to make ARBIN react properly. In our implementation, there was no 3D AR model placed at the destination. Therefore, we asked volunteers to check if ARBIN could guide them to the destinations successfully. The results show that ARBIN could successfully guide the volunteers to their destinations.

**Figure 15:**
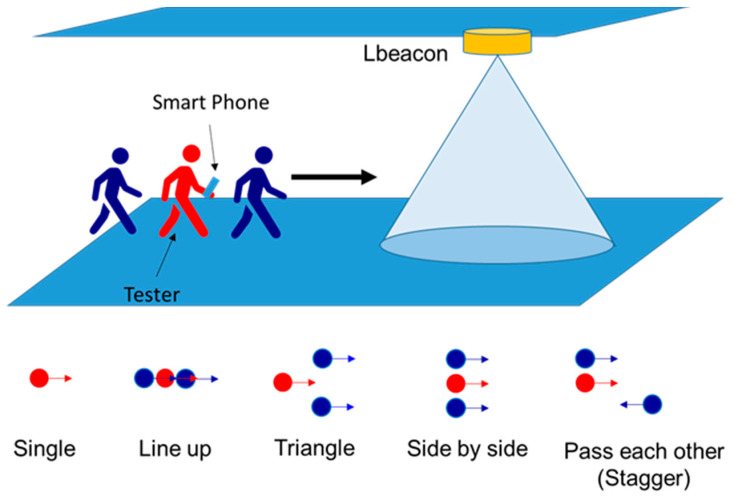
Different walking patterns of user.

In addition to the user experience evaluation, we also measured the responsiveness of ARBIN in the hospital. Samsung Galaxy S10e and Sony Xperia XZ Premium were used for the evaluation. We evaluated the responsiveness of the ARBIN along the route: Entrance Hall(A6) -> Registration counter(A11) -> Outpatient clinic(A22)-> X-ray examination room (B3) -> Pharmacy(A25) -> Exit (C1). There were in total 14 waypoints on the route. As shown in Figure 15, five different walking patterns of pedestrians were evaluated. The walking speed was around one meter per second. In our experiment, the area of a waypoint was set at a radius of 5 m. We used (Slow, Moderate, Fast) to represent responsiveness distance (~0 m, 0 m~5 m, >5 m). For example, if the responsiveness distance was larger than 5 m, we marked it as fast. No signal indicates that ARBIN had no response when the user entered the waypoint. We repeated the experiment five time, and summarize the results in Table 5. In the single mode and side-by-side mode, the responsiveness distance of both smartphones was always in the range of 0 m to 5 m at every waypoint we measured. In the triangle mode, the response distance of the Sony Xperia XZ Premium became slow at waypoint B3. Additionally, in the line-up mode and stagger mode, Sony Xperia XZ Premium had no response in waypoint B3 and A10. The possible reason may have been the poor design of antenna of the Sony Xperia XZ Premium. The situation could be improved by deploying one more Lbeacon at these waypoints to reduce the possibility of no reaction or slow reaction.

## 5. Conclusions

In this paper, we presented ARBIN, an augmented reality-based indoor navigation system, to guide users to their destinations in an indoor environment. When users enter the range of a waypoint, ARBIN posts a 3D directional indicator into the real-world surrounding environment. With the support of augmented reality, it is easier for users to determine their locations when walking inside a building. To address the heterogeneous problems of Lbeacons, four types of RSSI model are proposed. Experiences in correctly placing a 3D model in a real-world were also explained. Further, we conducted both in-house experiments and a field trial to verify the responsiveness and practicality of ARBIN. The in-house experiments showed that in 92.5% of the test cases, ARBIN could provide users with a proper directional indicator when they came close to a Lbeacon. For the field trial, four volunteers were invited. Of the of the user feedbacks, 97% (35/36) were moderate. Our results show that ARBIN can achieve a 3 to 5 m accuracy, and provide users with correct instructions on their way to the destinations. ARBIN proved to be a practical solution for indoor navigation, especially for large buildings. To further enhance user experience, in the future we plan to extend the capability of ARBIN by adding landmark objects into real-world environments, and showing advertisement messages provided by a surrounding information system.

## Figures and Tables

**Table 1:** The smartphones used for the experiment.

Model	CPU	Memory	OS	Bluetooth
Samsung Galaxy S10e	Exynos 9820	6 GB	Android 9.0	5.0
Sony Xperia XZ Premium	Snapdragon 835	4 GB	Android 9.0	5.0

**Table 2:** The accuracy of azimuth.

Azimuth (Degree)	Samsung S10e		SONY Xperia XZ Premium	
	Max (Degree)	Min (Degree)	Max (Degree)	Min (Degree)
North (0)	+2.66	–3.32	+2.35	–1.46
Northeast (45)	+4.67	–1.35	+0.98	–2.18
East (90)	+3.26	–2.54	+1.23	–3.35
Southeast (135)	+2.09	–2.52	+2.33	–3.04
South (180/–180)	–0.03	+0.01	–0.01	+0.01
Southwest (–135)	+1.94	–3.48	+1.74	–3.05
West (–90)	+2.28	–3.77	+3.44	–4.31
Northwest (–45)	+2.49	–3.64	+1.28	–1.65

**Table 3:** The results of responsiveness distance.

Lbeacon	Samsung Galaxy S10e		Sony Xperia XZ Premium	
	L		L	
	Forward Direction	Backward Direction	Forward Direction	Backward Direction
A1	2.0 m	0.8 m	0.2 m	0.4 m
A2	2.0 m	1.1 m	0.3 m	0.4 m
A3	3.0 m	5.3 m	1.4 m	0.4 m
B1	2.1 m	1.4 m	1.1 m	0.4 m
B2	1.0 m	3.1 m	2.2 m	0.6 m
B3	0.1 m	1.8 m	0.2 m	0.2 m
B4	2.2 m	2.0 m	1.2 m	0.2 m
C1	1.5 m	1.6 m	0.5 m	1.2 m
C2	1.3 m	3.1 m	1.1 m	1.2 m
C3	2.7 m	3.0 m	1.5 m	1.9 m
Average	1.7 m	2.3 m	1.0 m	0.7 m

**Table 4:** The volunteers’ feedback on responsiveness.

Task 1: Go to The Register Counter and Then the Clinic.					
Route Information		Volunteer A (Samsung)	Volunteer B (Asus)	Volunteer C (Oppo)	Volunteer D (SONY)
Actions	Lbeacons				
Go to the registration counter	A6–A11	Moderate	Moderate	Moderate	Moderate
Go to the clinic	A11–A20	Moderate	Moderate	Moderate	Moderate
	A20–A22	Arrive successfully	Arrive successfully	Arrive successfully	Arrive successfully
**Task 2: Go to the X-ray examination room.**					
Go to the stairs	A22–A20	Moderate	Moderate	Moderate	Moderate
	A20–A19	Moderate	Moderate	Moderate	Moderate
	A19–A18	Moderate	Moderate	Moderate	Moderate
Down the stairs	A18–B1	Moderate	Moderate	Moderate	Moderate
Go to the examination room	B1–B3	Arrive successfully	Arrive successfully	Arrive successfully	Arrive successfully
**Task 3: Go to the pharmacy.**					
Go to the stairs	B3–B1	Moderate	Moderate	Moderate	Moderate
Go up the stairs	B1–A18	Moderate	Slow	Moderate	Moderate
Go to the pharmacy	A18–A25	Arrive successfully	Arrive successfully	Arrive successfully	Arrive successfully
**Task 4: Go to the exit.**					
Go to the exit	A25–A31	Moderate	Moderate	Moderate	Moderate
	A31–C1	Arrive successfully	Arrive successfully	Arrive successfully	Arrive successfully

**Table 5:** The volunteers’ feedback on responsiveness.

Model/Working Pattern		No Signal	Slow	Moderate	Fast
Samsung Galaxy S10e	Single	0	0	14	0
	Line up	0	0	14	0
	Triangle	0	0	14	0
	Side by side	0	0	14	0
	Stagger	0	0	14	0
Sony Xperia XZ Premium	Single	0	0	14	0
	Line up	1 (B3)	0	13	0
	Triangle	0	1 (B3)	13	0
	Side by side	0	0	14	0
	Stagger	1(A10)	0	13	0
